# *In Vivo* Mouse Intervertebral Disc Degeneration Model Based on a New Histological Classification

**DOI:** 10.1371/journal.pone.0160486

**Published:** 2016-08-02

**Authors:** Takashi Ohnishi, Hideki Sudo, Koji Iwasaki, Takeru Tsujimoto, Yoichi M. Ito, Norimasa Iwasaki

**Affiliations:** 1 Department of Orthopaedic Surgery, Hokkaido University Graduate School of Medicine, Sapporo, Hokkaido, Japan; 2 Department of Advanced Medicine for Spine and Spinal Cord Disorders, Hokkaido University Graduate School of Medicine, Sapporo, Hokkaido, Japan; 3 Department of Biostatistics, Hokkaido University Graduate School of Medicine, Sapporo, Hokkaido, Japan; University of Pennsylvania, UNITED STATES

## Abstract

Although human intervertebral disc degeneration can lead to several spinal diseases, its pathogenesis remains unclear. This study aimed to create a new histological classification applicable to an *in vivo* mouse intervertebral disc degeneration model induced by needle puncture. One hundred six mice were operated and the L4/5 intervertebral disc was punctured with a 35- or 33-gauge needle. Micro-computed tomography scanning was performed, and the punctured region was confirmed. Evaluation was performed by using magnetic resonance imaging and histology by employing our classification scoring system. Our histological classification scores correlated well with the findings of magnetic resonance imaging and could detect degenerative progression, irrespective of the punctured region. However, the magnetic resonance imaging analysis revealed that there was no significant degenerative intervertebral disc change between the ventrally punctured and non-punctured control groups. To induce significant degeneration in the lumbar intervertebral discs, the central or dorsal region should be punctured instead of the ventral region.

## Introduction

Human intervertebral disc (IVD) degeneration is a common cause of low back pain and it affects the daily activities [1–3]. It is the cause of spinal diseases such as spinal canal stenosis, disc herniation, and spinal deformity. Currently, there is no clinical treatment to prevent the development of IVD degeneration, and the present available therapeutic options for spinal complications, namely analgesics and surgical procedures, do not address the etiology [1].

The research focusing on IVD degeneration, from the gross anatomical to histological studies, has been conducted using various animal models: scalpel incision to annulus fibrosus (AF) in canines [4] and rats [5]; surface incision to AF in sheep [6]; full puncture to IVD using needle in mice[7] and rats [8]; and hemi-AF puncture using needle in mice [9], rats [10], and rabbits [11–13]. However, because of the animal size and the species-specific physiological variations, the level of IVD degeneration differs [10,14]. To establish an IVD degeneration model induced by needle puncture, a precise size and shape of the device and a detailed procedure for each of the species are necessary. In addition, although the number of reports describing IVD degeneration in terms of the genetic approaches is increasing [1,15–19], there has been no appropriate histological classification applicable to an *in vivo* mouse intervertebral disc degeneration model, which is also applicable to genetically modified mice. The aim of this study was to create a new histological classification applicable to an *in vivo* mouse intervertebral disc degeneration model induced by needle puncture.

## Materials and Methods

All animal procedures in this study were conducted with the approval of the Institutional Animal Care and Use Committee of Hokkaido University (approval number: 13–0051). Moreover, all these procedures were carried out in accordance with the approved guidelines. Inbred C57BL/6 mice were obtained from Sankyo Labo Service Corporation (Tokyo, Japan). The mice were bred and housed under specific pathogen-free conditions before surgery, and housed under P2 conditions after surgery at Hokkaido University Creative Research Institution Platform for Research on Biofunctional Molecules. P2 means a level of isolation from natural environment, that keeps the room in sterilized and safe condition. They were kept in cages at room temperature (23°C ± 2 °C) and humidity of 50% ± 10%, under standard laboratory conditions with a 12 h light/dark cycle. They were allowed unrestricted cage activity and ad libitum access to food and water. Standard laboratory diet, Labo MR Stock (Nosan Corporation, Yokohama, Japan) and sterilized tap water were provided as sources of food and water, respectively. All the surgeries were performed under general anesthesia with ketamine 1.9 mg and xylazine 0.2 mg intraperitoneal injection, and all animal suffering was minimized. After surgery, the mice were monitored once in two days. During the experiment, five mice (which are not included in the total of 106 mice) died within one to two days after surgery, presumably with respiratory depression due to anesthesia or hemorrhage due to surgery. The protocol of early euthanasia/humane endpoints for mice that were severely ill or moribund, as indicated by shivering and respiratory distress with disability in walking suggestive of apparent distress, with no recovery expected, was intraperitoneal injection of 5 mg of pentobarbital sodium. However, none was applicable. Except for the five mice that died, all mice exhibited good health and well-being until the end of the experiment. They were euthanized with pentobarbital sodium intraperitoneal injection.

**Fig 1:**
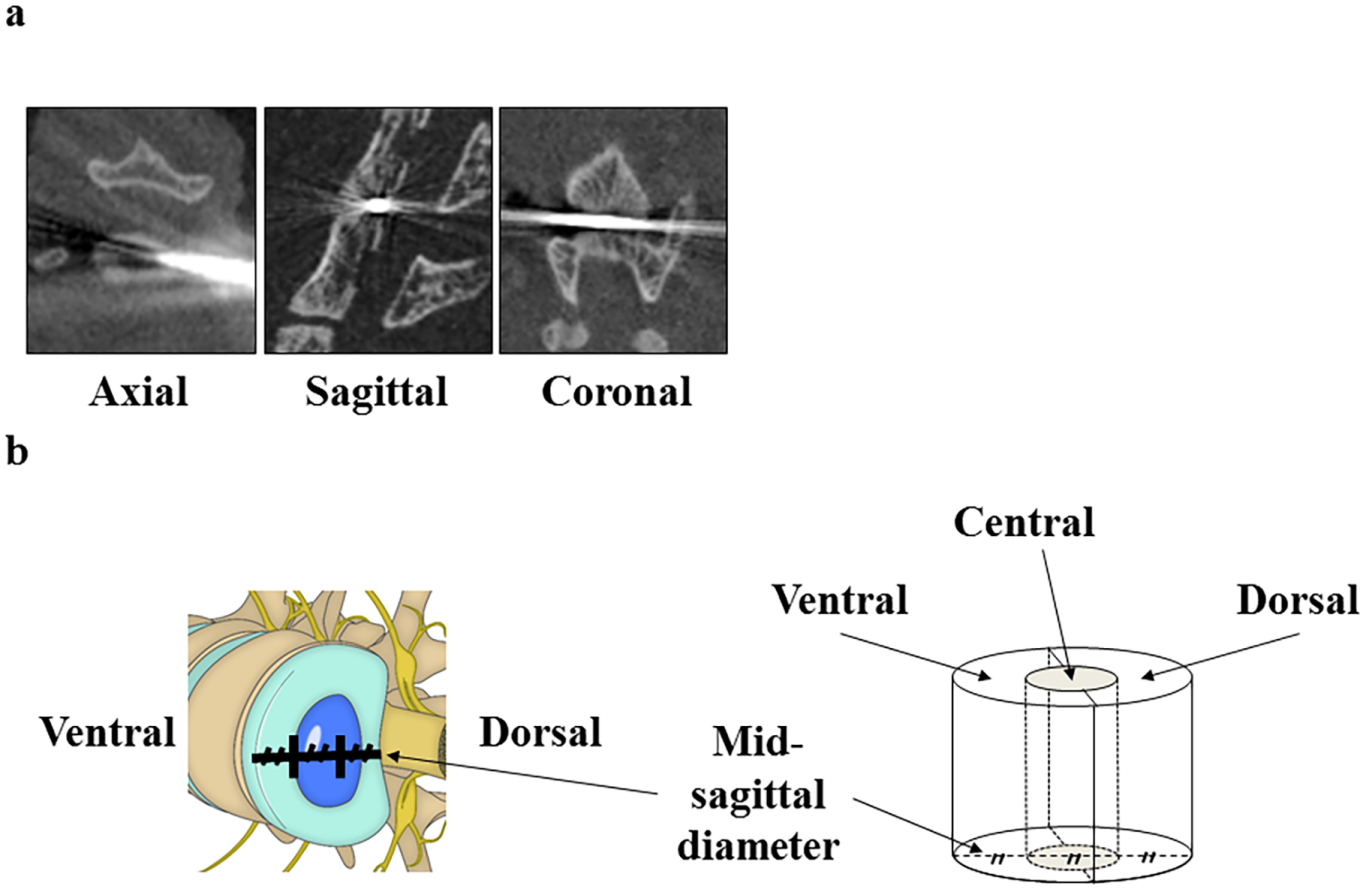
The location of a needle in the intervertebral disc was evaluated using micro-computed tomography scanning. (**a**) The punctured region was confirmed by multiplanar reconstruction views. (**b**) The punctured region was defined as the ventral (V), central (C), or dorsal (D) region.

One hundred six C57BL/6 mice (male, 54; female, 52) were operated under general anesthesia. All the mice were 11 weeks old at the time of surgery. The lumbar spine was posterolaterally approached from the right side, and the L4/5 IVD was punctured with a 35-gauge (G) or 33G needle. Micro-CT scanning [3D micro X-ray CT R_mCT 2 (Rigaku, Tokyo, Japan)] was performed, and the punctured region was confirmed through multiplanar reconstruction views (Fig 1a). The regions in IVD were determined in order to identify the position of the needle, which are as follows. First, the mid-sagittal diameter was divided into three parts: a concentric ellipsoid that constituted the central region was described using boundary points. Second, the peripheral region was divided into the ventral and dorsal regions. Third, the ventral, central, and dorsal regions were defined (Fig 1b). The regions were space prescribed by the endplates. The outer layer of AF that bulged from the endplates was not counted as the intervertebral space. When the needle did not get punctured into the intervertebral space, the trial was repeated until the needle hit the intervertebral space. We determined the position of the needle by visually analyzing the multi-slice CT scan images. If the needle penetrated the central region, we designate the position of the needle as the ‘central region’, and in cases when the needle partially penetrated the dorsal or ventral region, consensus was obtained for the designation of the region. Before closing the wound, the needles were removed and the mice were euthanized one, two, four, eight, or 12 weeks after the surgery.

The mid-sagittal images of the punctured discs were qualitatively analyzed by using MRI to evidence the degenerative changes. More precisely, T2-weighed mid-sagittal images of the punctured discs were qualitatively analyzed by using a 7.0-Tesla MR scanner (Varian Unity Inova; Varian Medical Systems, Palo Alto, CA, USA) [1,17,20]. The degree of IVD was assessed by using the Pfirrmann classification [21]. A quantitative analysis of the sagittal image slices was also performed by using Analyze 10.0 software (AnalyzeDirect, Overland Park, KS, USA), as reported previously [1,17,20]. To quantify the alterations in the NP, the MRI index (the product of the NP area and the average signal intensity) was used [1,17,20]. Data were expressed as percentages of the results obtained when using untreated, non-punctured control discs [1,17,20]. The control was defined for the Pfirrmann grade as the value of L3/4 IVD and for the MRI index as the average value of both L3/4 and L5/6 IVDs. All the image assessments were performed by two independent blind observers, and the quantitative data were presented as means of three evaluations.

**Fig 2:**
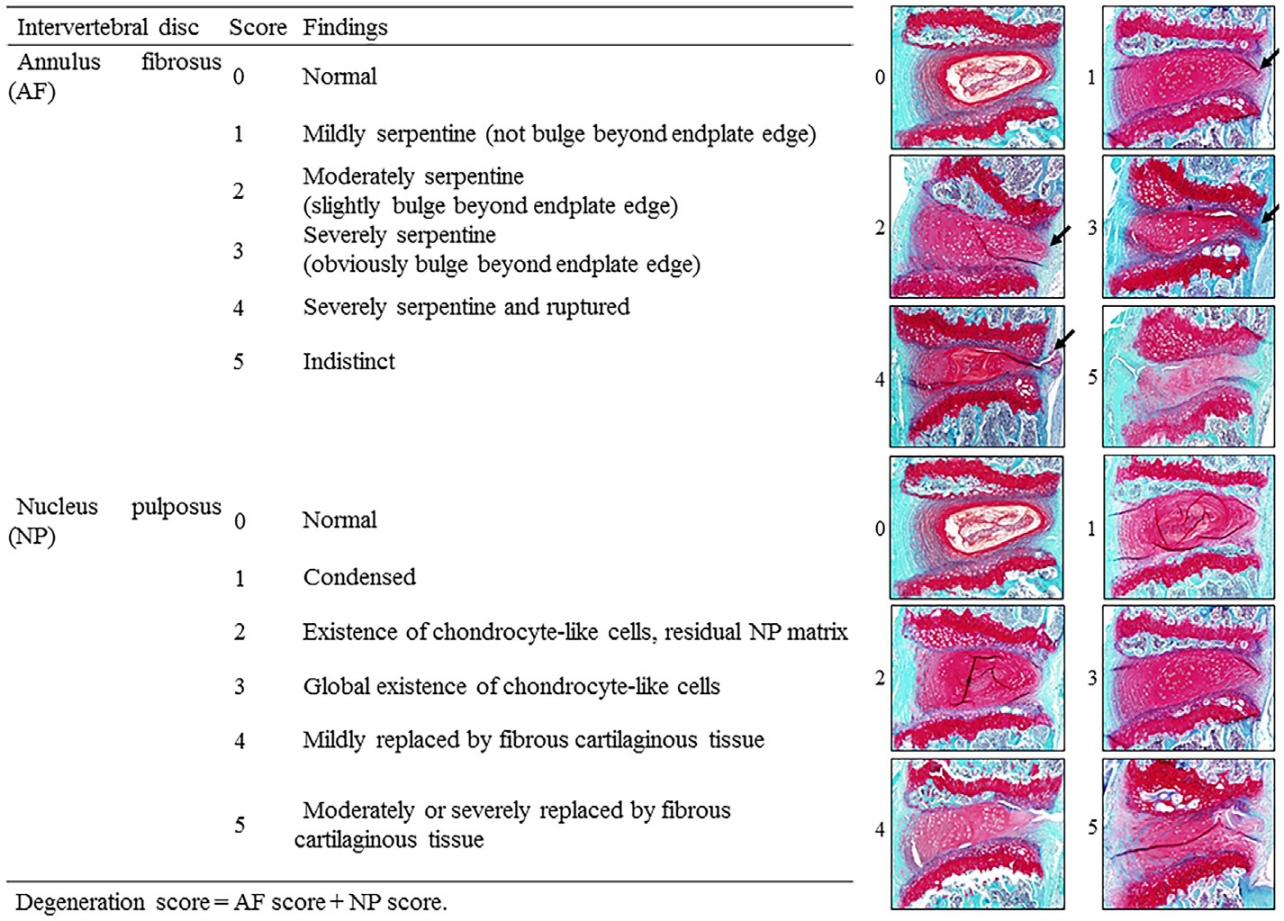
The novel proposed histological grading score. Arrows indicate the serpentine findings in the annulus fibrosus.

After the MRI examinations, each IVD was fixed in 10% neutral buffered formalin solution for 48 h, followed by decalcification with 10% EDTA for 2–4 weeks, and paraffin embedding. Mid-sagittal sections were obtained and stained with safranin O-fast green. For the histological analysis, four types of classification [for rabbits by Masuda et al. [11], for rats by Nishimura et al. [22], for mice by Yang et al. [9], and our group (Fig 2)] were used to evaluate the degeneration. For each classification type, the maximum points represent severe degeneration. The control was defined as the histological score of L3/4 IVD. All the histological assessments were performed by two independent blind observers, and the quantitative data were presented as the mean of three evaluations. Our internal studies of intra-/inter-rater reliability have shown excellent kappa statistics for all measures regarding MRI and histological examinations (0.85–1.0).

**Fig 3:**
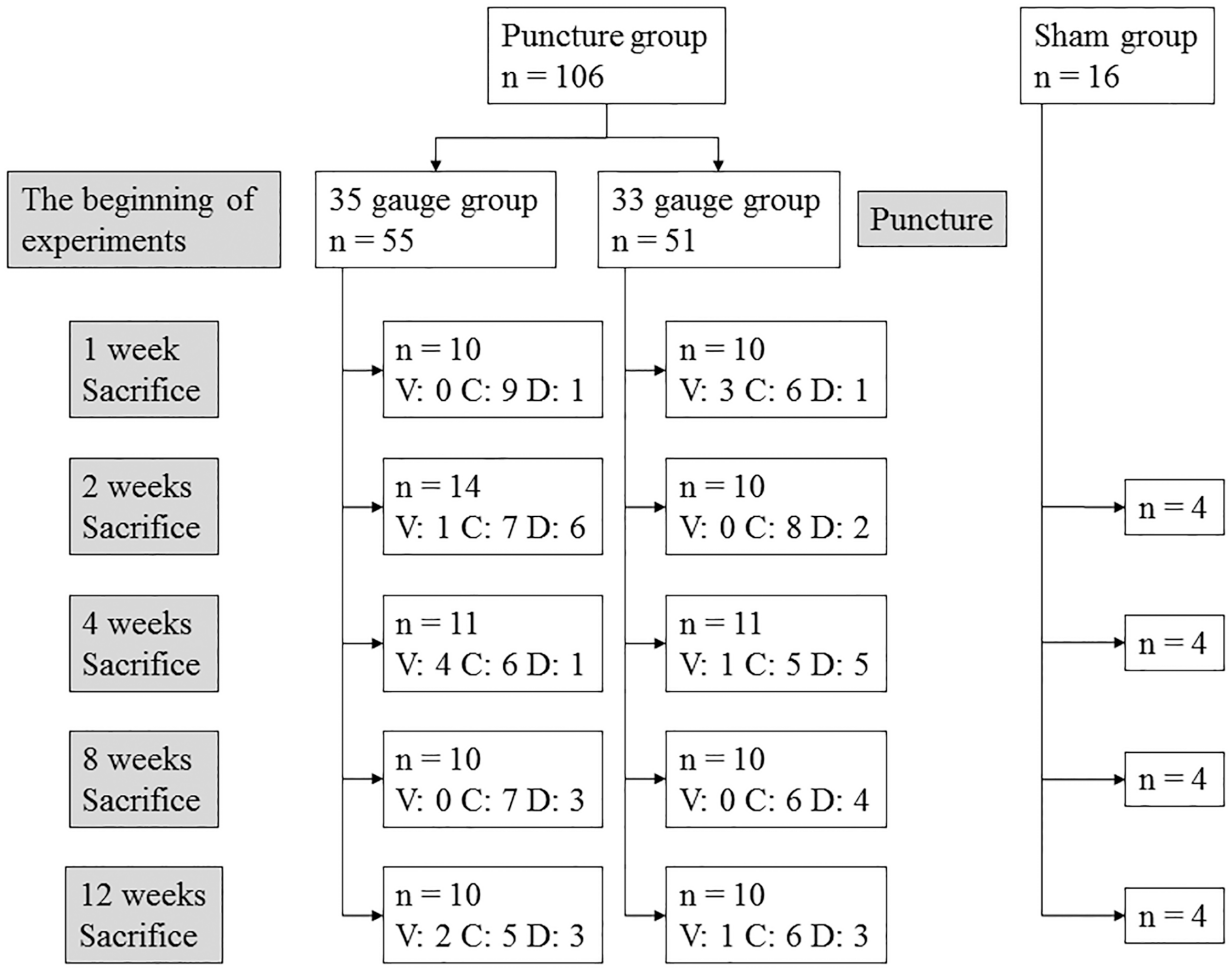
The flowchart of the experiment indicating the time course and the case number. V, ventral region; C, central region; D, dorsal region.

The number of mice for each needle size and time point were as follows; ten for the 1-week, fourteen for the 2-week, eleven for the 4-week, ten for the 8-week, and ten for the 12-week time point for the 35G needle puncture; ten for the 1-week, ten for the 2-week, eleven for the 4-week, ten for the 8-week, and ten for the 12-week time point for the 33G needle puncture. A sham operation was defined as the posterolateral surgical approach without a needle puncture. The number of mice stratified according to sex in each time point of the 35G group were as follows: 1-week: female, 5; male, 5; 2 weeks: female, 10; male, 4; 4 weeks: female, 7; male, 4; 8 weeks: female, 5; male, 5; 12 weeks: female, 5; male, 5; for the 33G group: 1 week: female, 5; male, 5; 2 weeks: female, 1, male, 9; 4 weeks: female, 6; male, 5; 8 weeks: female, 5; male, 5; 12 weeks: female, 3; male, 7. For each time point there were four sham operations (female, 2; male, 2) (Fig 3).

Furthermore, statistical analyses were performed. A correlation analysis was used to evaluate the MRI index relationships with the histological classification scores. The multiple regression analysis was used to determine whether the postoperative time points and the punctured regions were significant variables. Kruskal-Wallis test was used for comparison of each subgroup of region with non-punctured control. Single regression analysis was used to determine whether the postoperative time point is a significant variable for NP score or AF score of punctured IVD. The Tukey HSD test was used for comparison of NP score at each time point. Single regression analysis was used to determine whether the postoperative time point is a significant variable for IVD height and width of punctured IVD. The Kruskal Wallis test was used to compare the 35G and the 33G needle for Pfirrmann grades, MRI indexes, or our histological classification scores.

## Results

### The comparison of four histological classification scores

**Fig 4:**
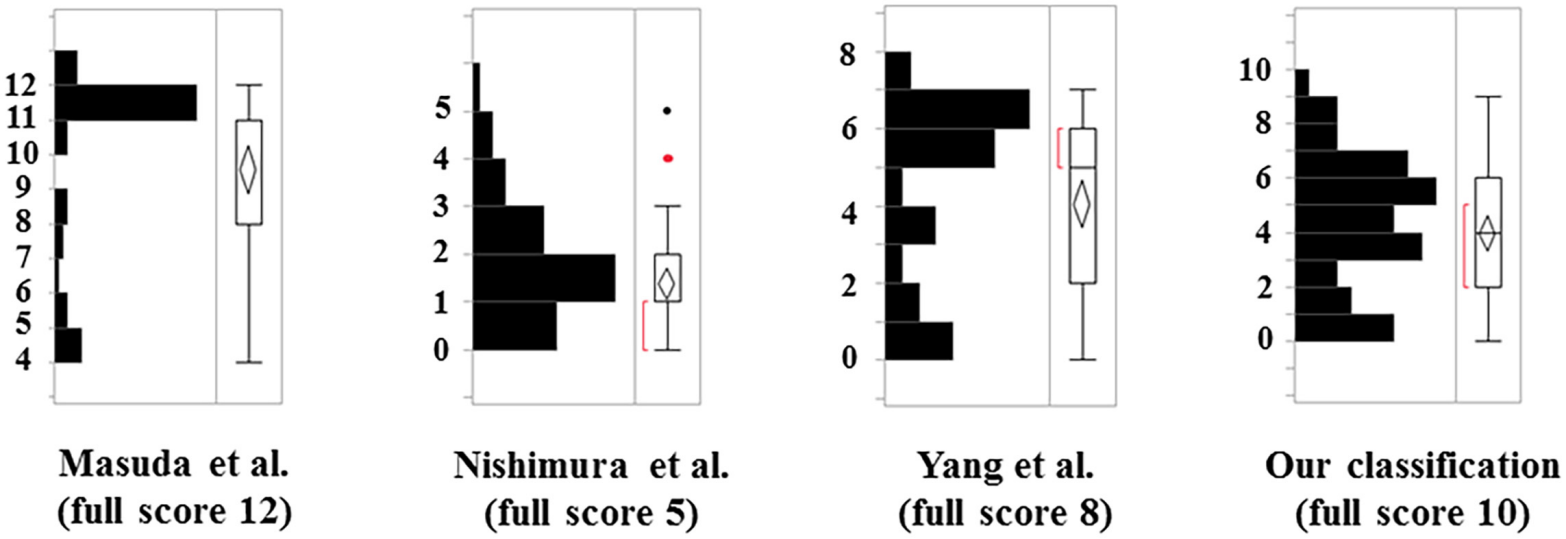
The comparison of the histological classifications. The distribution of scores in each classification. Red brackets indicate the minimum ranges that include 50% of the data. The red and black dots of the classification score of Nishimura et al.[22] indicate the outliers.

We firstly analyzed the score distribution according to four histological classifications. Fig 4 shows the data distributions of the 35G puncture group based on the histological classification scores. The score distribution of the classification by Masuda et al. [11] did not show normality, with a skewness (Sk) value of -1.27. Additionally, the score showed a ceiling effect, indicating that it was inappropriate for the present mice IVD degeneration model. In contrast, the distribution of the scores by Nishimura et al. [22]’s, Yang et al. [9]’s, and our classifications showed normality, given that their Sk and kurtosis (Ku) were less than 1.00 (Sk = 0.97, Ku = 0.66 by Nishimura et al. [22], Sk = -0.69, Ku = -0.96 by Yang et al. [9], and Sk = -0.09, Ku = -0.72 by our classification). However, the Sk of Nishimura et al. [22]’s classification was higher than ours because their score had some outliers. In addition, compared to our classification, the Nishimura et al. [22] score showed a floor effect and the Yang et al. [9] score showed a ceiling effect. These results indicated that our classification could more precisely detect the gradual progression of the degenerative changes (Fig 4).

### Correlation analysis relating MRI index

**Fig 5:**
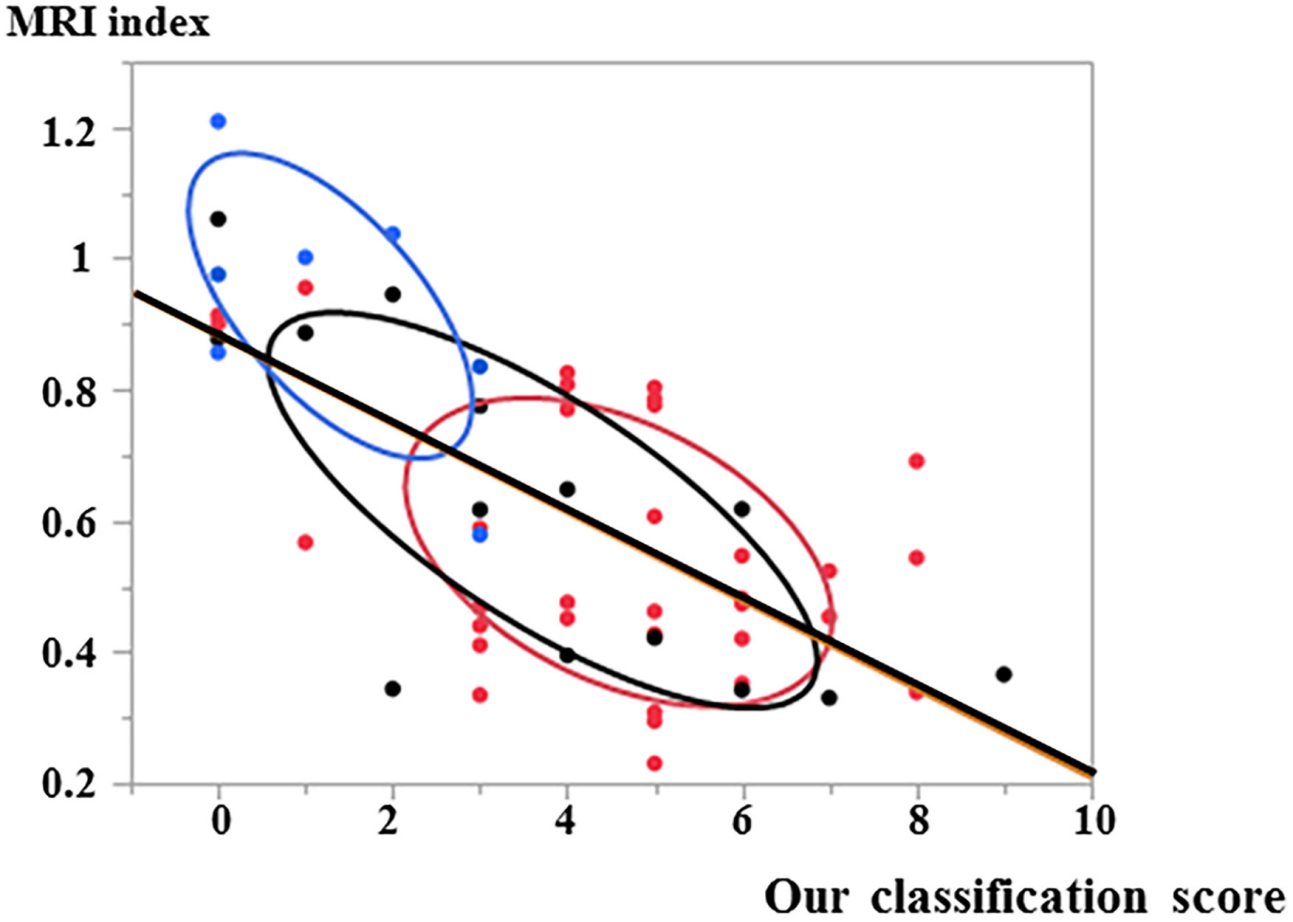
Correlation analysis between our histological score and MRI index. The blue, black, and red dots indicate the ventral, dorsal, and central region cases, respectively. Ovals indicate 50% confidence ellipse with bivariate normal distribution of each region.

In the correlation and the simple linear regression analysis relating the MRI index with our classification, the results yielded ρ = -0.66 and R^2^ = 0.44, showing that our classification yielded a good correlation and linear fit with the MRI index (Fig 5).

In a scatterplot and probability ellipse analysis, the 50% confidence probability ellipse for each punctured region were thin and well separated, indicating the good linear fit of our score. In addition, our score showed significant correlation with the MRI index in the central and dorsal regions with P values of 0.01 and 0.002, respectively (Fig 5).

### Significant variable for inducing degenerative IVDs

Next, to identify the variables most predictive of degenerative IVDs, the following were tested in the multiple regression analysis: punctured region (including ventral, central, and dorsal regions) and time point. The results of the analysis revealed that not the “time point” but the “punctured region” was predictive of the degenerative outcomes (Table 1).

**Table 1:** Multiple regression analysis to identify significant variable for the degenerative intervertebral discs.

	Punctured region	Time point
35G		
Pfirrmann grade	< 0.01*	0.99
MRI index	< 0.01*	0.03**
Classification by us	0.04*	0.20
33G		
Pfirrmann grade	< 0.01*	0.34
MRI index	< 0.01*	0.13
Classification by us	< 0.01*	0.27

Numerical values indicate p values.

*Statistically significant.

**Not significant as a result of Tukey HSD test.

**Fig 6:**
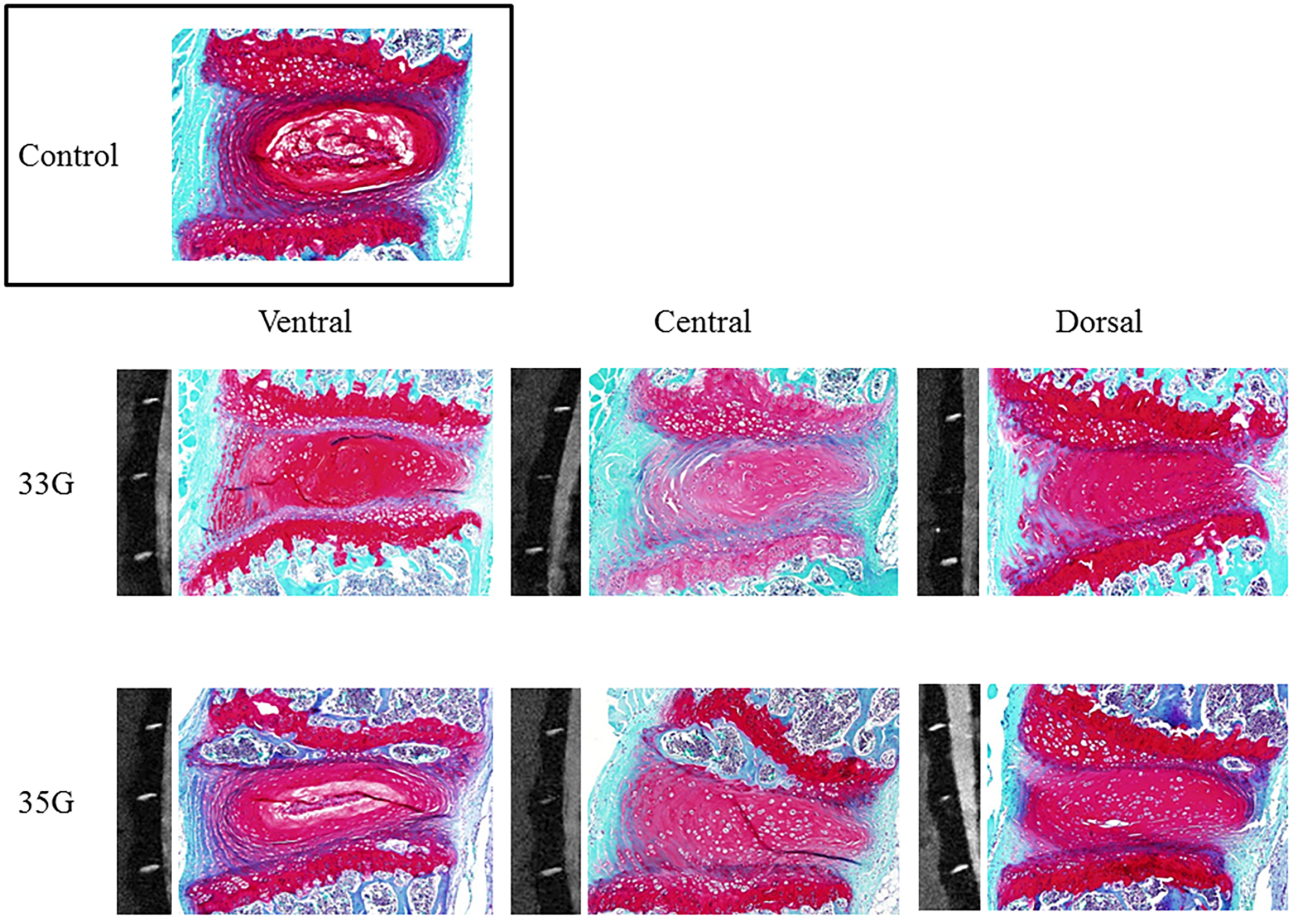
Punctured region and intervertebral disc degeneration. Representative magnetic resonance imaging and histological images at four weeks after puncturing with 35- and 33-gauge needles.

We further analyzed the whole data regarding punctured region. Compared with the non- punctured IVDs, those punctured through the central or the dorsal region showed significantly higher Pfirrmann grade and lower MRI index. Similarly, on using our classification, the IVDs punctured through the central or the dorsal region showed significantly higher histological scores. For the IVDs punctured through the ventral regions, our histological score was significantly higher compared with that for the non-punctured IVDs. However, in the MRI analysis, no significant degenerative IVD difference was observed between the ventrally punctured and the non-punctured control groups (Table 2) (Fig 6).

**Table 2:** Punctured region and intervertebral disc degeneration compared to the control.

	Central region	Dorsal region	Ventral region
35G			
Pfirrmann grade	> Control*	> Control *	= Control
MRI index	< Control *	< Control *	< Control
Our classification score	> Control *	> Control *	> Control *
33G			
Pfirrmann grade	> Control *	> Control *	> Control
MRI index	< Control *	< Control *	< Control
Our classification score	> Control *	> Control *	> Control *

*p < 0.05.

In addition to the total score, AF and NP scores were separately analyzed. The single regression analysis was used to determine whether the postoperative time point was significant variable for NP score or AF score of punctured IVD excluding ventral puncture. For comparisons of NP score of each time point, Tukey HSD test was used. There was no significant change in the AF and NP scores, except that both 8- and 12-week NP scores were significantly higher compared to 1-week NP score in the 33G needle puncture group (data not shown). As for IVD height and width, the single regression analysis was also used to determine whether the postoperative time point is significant variable for IVD height and width of punctured IVD excluding ventral puncture. The L4/5 IVD punctured with either the 35G or the 33G needle showed an approximately 10% decrease in IVD height and approximately 20% (35G) or 30% (33G) increase in IVD width 1 week after the puncture compared to L3/4 non-punctured control IVD. However, there was no significant difference in IVD height and width among each time point (data not shown).

### Center of the nucleus pulposus (NP) was deviated dorsally in IVD

**Fig 7:**
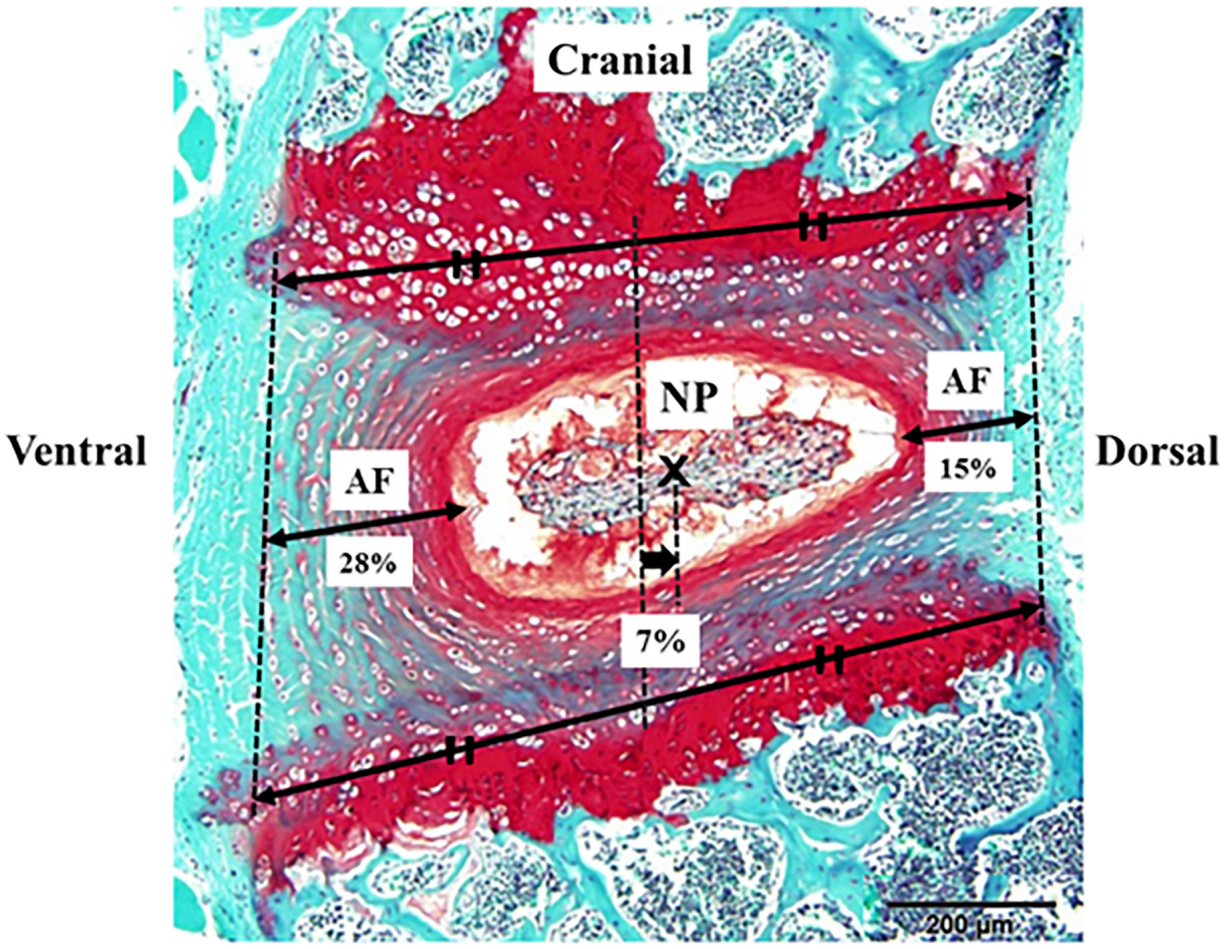
The mid-sagittal slice of the lumbar intervertebral disc (IVD). The nucleus pulposus (NP) center was deviated dorsally relative to the IVD center. In 77 intact mouse lumbar IVDs, the width of ventral annulus fibrosus (AF), the dorsal deviation of the NP center relative to the IVD center, and the width of dorsal AF were calculated as proportion relative to the major axis of the IVD. The average amount of NP deviation (7%) corresponded to the difference in width between the average ventral (28%) and the average dorsal AF (15%): the ventral site of AF is thicker compared to the dorsal site.

From the anatomical point of view, we measured the deviation of the NP center relative to the IVD center in 77 intact mouse lumbar IVDs. To match the results of the punctured regions based on the CT scan images and histological evaluations, the AF was measured on the space prescribed by the endplates. After the NP position was determined visually, the measurement was performed using Image J software (National Institutes of Health, Bethesda, Maryland, USA). The width of ventral AF, the dorsal deviation of the NP center relative to the IVD center, and the width of the dorsal AF were calculated as proportion relative to the major axis of the IVD. The average amount of the NP deviation (7%) corresponded to the difference in width between the average ventral (28%) and average dorsal AF (15%): the ventral site of AF is thicker compared to the dorsal site (Fig 7).

### Intervertebral disc degeneration and needle size

Based on either the MRI (Table 3) or the histological analysis by our classification (Table 4), the IVD punctured with the 33G needle showed more degenerative changes compared with the IVDs punctured with the 35G needle. Regarding the vascularization and mineralization of the end plate, there was no certain findings in this puncture model (data not shown). In the sham group, both the MRI and histological findings were normal.

**Table 3:** Intervertebral disc degeneration and needle size.

	35G	33G	P
Pfirrmann grade			
1-week	2.50 ± 0.71	3.71 ± 1.11	0.03*
2-week	2.54 ± 0.88	4.30 ± 0.82	< 0.01*
4-week	2.43 ± 1.13	4.50 ± 0.53	< 0.01*
8-week	2.50 ± 0.85	4.60 ± 0.70	< 0.01*
12-week	2.50 ± 0.93	4.22 ± 0.83	< 0.01*
MRI index			
1-week	0.71 ± 0.14	0.47 ± 0.11	< 0.01*
2-week	0.54 ± 0.23	0.34 ± 0.09	0.01*
4-week	0.62 ± 0.29	0.34 ± 0.09	0.04*
8-week	0.51 ± 0.17	0.34 ± 0.15	0.02*
12-week	0.49 ± 0.20	0.34 ± 0.10	0.08

Ventral puncture data were removed from groups.

*Statistically significant.

**Table 4:** Intervertebral disc degeneration and needle size.

	35G	33G	P
Our classification score			
1-week	5.10 ± 1.91	4.86 ± 1.57	0.73
2-week	4.15 ± 1.82	6.30 ± 2.83	0.08
4-week	4.00 ± 3.00	6.70 ± 2.54	0.11
8-week	5.20 ± 2.66	7.30 ± 1.25	0.05*
12-week	2.88 ± 1.73	7.11 ± 1.69	< 0.01*

Ventral puncture data were removed from groups.

*Statistically significant.

## Discussion

Although it is ideal to use an age-related IVD degeneration mouse model to investigate the mechanisms of IVD degeneration, some genetically modified mice have short life spans [23,24]. To overcome this limitation, an *in vivo* mouse IVD degeneration model induced by needle puncture is needed. There may be a criticism that the IVD of a quadruped animal was used as an alternative to the bipedal human IVD. However, Elliott et al. [25] reported that the mouse discs, when normalized for geometry, they represented well the mechanical properties of the human lumbar spine. Their findings provided strong support for the use of the rodent model in the study of human disc function, disease, and degeneration. In addition, they found that some geometrical and mechanical properties correlated with the animal body weight in the lumbar spine but no parameter correlated in the tail spine, suggesting that the lumbar spine is a more appropriate model of the bipedal human spine than is the tail spine. [25,26] This is because the loading from the animal body weight may be transferred to the rodent lumbar spine.

To our knowledge, there is only one report of mouse lumbar IVD degeneration model induced by needle puncture [27]. However, there were some limitations to that study: short duration of follow-up, small number of cases, and subjective histological evaluation [27]. In addition, they punctured consecutive three discs in one mouse (L4/5, 5/6, 6/S1), and they evaluated the punctured status by using naked eye observation only [27]. In contrast, in our study, the number of mice was greater than 10 for each time point, and the follow-up periods were of up to 12 weeks. In addition, we used four histological classification scores. Only one lumbar IVD per mouse was punctured, and the punctured status was verified by CT scanning.

Masuda et al. [11] were the first to establish a rabbit model of disc degeneration based on puncturing the AF with a needle and evaluating the IVD degeneration by their original grading system for histology. Nishimura et al. [22] also evaluated IVD degeneration by their original grading system for rat IVD. Furthermore, Yang et al. [9] evaluated IVD degeneration by their original grading system for mice IVD. In the present study, the classification by Masuda et al. [11] showed a severe score even at early time points. In their classification, the AF grade received the maximum score when 30% of the AF fibers were serpentine, and the NP grade received the maximum score when NP was moderately condensed. In mice, those findings commonly appeared at the early stage or in mild degeneration. In the sections “border between the AF and NP” and “cellularity of the NP,” the same problem appeared. In the classification by Nishimura et al. [22], scores were condensed to 0 to 2 points. In their classification, NP was not evaluated. With reference to the mice, the degenerative changes in NP were drastic even in the early stage, meaning that by excluding the NP from the evaluation of IVD degeneration leads to underestimation. In the classification by Yang et al. [9], scores were condensed to 5 to 6 points, indicating that the classification could not differentiate severe degeneration. Consequently, neither classification could detect the gradual progression of the IVD degeneration. In contrast, our classification could classify precisely the gradual degenerative changes for mouse AF and NP, and combine them as a total degeneration score.

In the present study, by using our histological score, the punctured IVD showed significant degeneration irrespective of the punctured region. However, in the MRI analysis, there was no significant degenerative IVD change between the ventrally punctured and the non-punctured control groups. We also found that the ventral site of AF is thicker than the dorsal site. Thus, the biomechanical effects of needle puncture on IVD degeneration is thought to be less severe. Furthermore, the center of NP was deviated dorsally in IVD. NP plays central role in the biological reaction in the IVD [1,17]. Due to these anatomical characteristics and subsequent biomechanical/biological reasons, the degenerative change in the IVDs punctured through the ventral region was significantly milder compared to those of the IVDs punctured through the central or the dorsal region. To expand the versatility of the evaluation for IVD degenerative change to the MRI analysis, we recommend that the central or dorsal region be punctured.

Based on previous reports [14,27], we established the needle sizes as 35G and 33G. According to Elliot et al. [14], the ratio of the needle diameter to the punctured IVD height needed to exceed 0.4 to induce significant degeneration. Thus, in order to not destroy the IVD by a single puncture and induce subsequent degeneration, 35G (ratio 0.5) and 33G (ratio 0.87) were considered as reasonable diameters. Considering the severity of IVD degeneration, we recommend the use of the 35G model for the study of the IVD degeneration adjustment and the 33G model for the study of the IVD regeneration.

The present results also showed that the “time point” was not a predictive variable of the degenerative outcomes. Accordingly, the differences in Pfirrmann grades, the MRI indexes, and our histological classification scores among the 1-, 2-, 4-, 8-, and 12-week points were not significant. Therefore, we concluded that the mouse IVD degeneration induced by needle puncture progressed drastically in one week and then plateaued. This result suggests that one to two weeks of follow-up is sufficient for evaluating the degenerative outcome of the punctured IVD.

In conclusion, this study investigated the IVD region by puncturing with a needle with micro-CT scanning and the severity of the degeneration. Puncturing through the ventral region may not induce efficient degeneration. To induce significant degeneration in the lumbar IVD, the central or dorsal region should be punctured. In any case, our classification can detect the gradual progression of the degenerative changes. Based on the present results, researchers may replace the micro-CT scanning with high-resolution fluoroscopy for assistance in puncturing the central or dorsal region.
